# Resilient Networks of Ant-Plant Mutualists in Amazonian Forest Fragments

**DOI:** 10.1371/journal.pone.0040803

**Published:** 2012-08-09

**Authors:** Heather A. Passmore, Emilio M. Bruna, Sylvia M. Heredia, Heraldo L. Vasconcelos

**Affiliations:** 1 Department of Wildlife Ecology & Conservation, University of Florida, Gainesville, Florida, United States of America; 2 Center for Latin American Studies, University of Florida, Gainesville, Florida, United States of America; 3 Department of Botany and Plant Sciences, University of California Riverside, Riverside, California, United States of America; 4 Instituto de Biologia, Universidade Federal de Uberlândia, C.P. 593, Uberlândia, Minas Gerais, Brazil; 5 Biological Dynamics of Forest Fragments Project, Instituto Nacional de Pesquisas da Amazônia & Smithsonian Tropical Research Institute, Manaus, Amazonas, Brazil; Centro de Investigación y de Estudios Avanzados, Mexico

## Abstract

**Background:**

The organization of networks of interacting species, such as plants and animals engaged in mutualisms, strongly influences the ecology and evolution of partner communities. Habitat fragmentation is a globally pervasive form of spatial heterogeneity that could profoundly impact the structure of mutualist networks. This is particularly true for biodiversity-rich tropical ecosystems, where the majority of plant species depend on mutualisms with animals and it is thought that changes in the structure of mutualist networks could lead to cascades of extinctions.

**Methodology/Principal Findings:**

We evaluated effects of fragmentation on mutualistic networks by calculating metrics of network structure for ant-plant networks in continuous Amazonian forests with those in forest fragments. We hypothesized that networks in fragments would have fewer species and higher connectance, but equal nestedness and resilience compared to forest networks. Only one of the nine metrics we compared differed between continuous forest and forest fragments, indicating that networks were resistant to the biotic and abiotic changes that accompany fragmentation. This is partially the result of the loss of only specialist species with one connection that were lost in forest fragments.

**Conclusions/Significance:**

We found that the networks of ant-plant mutualists in twenty-five year old fragments are similar to those in continuous forest, suggesting these interactions are resistant to the detrimental changes associated with habitat fragmentation, at least in landscapes that are a mosaic of fragments, regenerating forests, and pastures. However, ant-plant mutualistic networks may have several properties that may promote their persistence in fragmented landscapes. Proactive identification of key mutualist partners may be necessary to focus conservation efforts on the interactions that insure the integrity of network structure and the ecosystems services networks provide.

## Introduction

While there is much to be learned about the dynamics of mutualisms from the study of pair-wise interactions [1], there has been an upsurge of interest in how the analysis of multi-species networks can enhance our understanding of these pivotal interactions [2], [3]. A network-focused approach has revealed that networks of plant-animal mutualists tend to be highly nested – both generalist and specialist species tend to interact with generalists [4], [5], [6]. They are also likely to be built on weak and asymmetric links, meaning a plant species that is very dependent on a particular animal species is only weakly depended on by that animal species [4], [7]. Understanding these and other properties of network structure can not only provide insights into the assembly and evolution of species interactions, but can also provide unique insights into community responses to anthropogenic disturbances such as habitat loss and species extinctions [8], [9], [10].

Despite these advances, however, most studies of mutualist networks fail to consider the complexity of the landscape in which these communities are embedded [8], [9]. For instance, habitat fragmentation is a globally pervasive form of landscape alteration that could profoundly impact network topology [8], [9]. Recent modeling efforts support this hypothesis. In simulations Morales & Vazquez [11] found that networks in spatially explicit landscapes had lower connectance, higher asymmetry, and less-predictable interactions when species were aggregated and animal mobility was limited. In contrast, Fortuna & Bascompte [12] found there was a threshold of habitat loss at which mutualistic networks ultimately collapsed. Despite the major implications of these results for the maintenance of biodiversity and ecosystem services in fragmented landscapes, empirical evaluations of these predictions are limited. Sabatino *et al.* [13] compared pollination networks found in isolated hills embedded in an agricultural matrix and found that there was a strong positive effect of fragment area on species richness and link number, while Piazzon *et al.* [14] found topological differences between the epiphyte-tree networks found in old-growth and disturbed forests sites. It is particularly notable, however, that it is virtually unknown how fragmentation influences network structure in biodiversity-rich tropical ecosystems (although at least one study has been conducted in Chaco forest [15]). Because the vast majority of plant species there are dependent on mutualisms with animals [16], it is thought that changes in the structure of mutualist communities could lead to cascades of extinctions in these increasingly fragmented landscapes [17].

Mutualisms between ants and specialized plants known as myrmecophytes are a defining feature of tropical forests [18]. Plants from over 100 genera have leaf pouches, swollen petioles, hollow stems, or other ‘domatia’ in which a suite of obligately associated ant species establish colonies. Resident ants defend their host plants from herbivores or competitors, and the loss of ant colonies can results in severe defoliation or plant death [19], [20]. Since the pioneering work of Janzen [21], ant-plant communities have become model systems to study the ecology and evolution of mutualisms, mechanisms promoting species coexistence, and trophic cascades [18], [19].

We evaluated the effect of habitat fragmentation on the structure of mutualistic networks using the community of ant-plant partners found in an experimentally fragmented landscape in the central Amazon. By comparing these networks in continuous forest sites with those in forest fragments, we provide the first empirical test of the effects of habitat fragmentation on network structure that have been put forward in prior theoretical and empirical work. To do so, we begin by comparing the diversity of ants and plants in fragments and continuous forest. Because species diversity of both plants and animals decreases in tropical forest fragments [22], [23] we predicted that fragments will have fewer species of mutualist plants and ants (i.e., fewer nodes) than continuous forest sites. We then predict these changes will have the following influence on network structure. First, because highly specialized species are especially prone to extinction, connectance (i.e., the ratio of actual to possible links) will be higher in networks in fragmented sites [24]. This disproportionate loss of specialists is also why we predict that nestedness, specifically weighted-interaction nestedness [25], will not differ significantly between intact forest and fragments. This prediction stems from the observation that the loss of specialized species from fragments [26] has less of an effect on nestedness than the loss of well-connected generalists [27], [28], [29]. Finally, we tested the prediction that communities of ant-plant partners in fragments will be less “robust”, i.e., tolerant to the extinction of individual species, despite the increased connectance resulting from the loss of specialists [24].

## Materials and Methods

### Ethics Statement

All necessary permits were obtained for the described field studies. All research was conducted with the approval of Brazil's National Council of Scientific and Technological Development (CNPq, Permit Number 276/2005) and the Brazilian Institute of Environment and Renewable Natural Resources (IBAMA, Permit Number 226/2005).

### Study sites and data collection

We conducted our study in the lowland tropical forests of Brazil's Biological Dynamics of Forest Fragments Project (BDFFP; 2°30′S, 60°W). From October 2001 to February 2002, we conducted surveys of the comprehensively described ant-plant mutualist community [30] found in four of the BDFFP's experimentally isolated 1-ha forest fragments (FF) and four continuous forest (CF) sites [31]. The fragments were isolated in the early 1980s and have been maintained isolated by regularly clearing a 100 m band of secondary growth surrounding the fragments. The habitat is non-flooded lowland forest with a 30–35 m tall canopy, an understory dominated by stemless palms, and rugged topography ranging from 50–150 m in elevation. Soils in the sites are highly acidic and nutrient poor xanthic ferralsols with poor water retention capacity [32]. Annual rainfall ranges from 1,900–3,500 mm per year, and there is a pronounced dry season from June–October. Details of the BDFFP's design, history, and biology can be found in Bierregaard *et al.* [33].

In each of our eight study sites we established a 100 m×100 m plot in which we mapped all ant-plants and recorded the presence and identity of ant colonies in their domatia; vouchers of plants and ants were collected to confirm identifications. Here we analyze the network connections between the 12 myrmecophytes we recorded in our surveys and the 10 obligate ant species associated with them reported in [31]. Note that as in our prior work [31] we considered all *Azteca* species as a single taxon because of unresolved taxonomy of this group. We also pooled all *Pouruma* (Cecropiaceae) into a single taxon because of the difficulty in classifying individuals to species with floral characters and no individuals were fertile during our surveys. Finally, difficulty in distinguishing among juvenile *Tachigali* trees led us to exclude smaller individuals found in two CF plots (N = 4 individuals total) and two FF plots (N = 12 individuals), although all adult trees were readily identified.

### Analytical Methods

Hypotheses about network structure can be addressed using both qualitative and quantitative approaches. Qualitative metrics are calculated from binary interaction matrices while quantitative metrics include information on the frequency of individual interactions. Qualitative metrics are still commonly used to study networks, and hence they are useful for comparing the results of our work with those of other studies. However, the frequency of an interaction between species is an important measure of its strength and hence importance [4]. In addition, quantitative descriptors have been found to be more robust to variable sampling efforts than qualitative ones [34], [35]. We therefore use both qualitative and quantitative approaches to test our hypotheses. We calculated all metrics using the R package Bipartite [36], [37], with the exception of the frequency-based weighted nestedness estimator for which we used the program WINE [V 3.2, 25 see Table 1 for the formulas used for all metrics]. Finally, we pooled data from all plots in a habitat class to construct a summary network and calculate the same metrics of network structure (sensu [38]).

**Table 1:** Metrics used to compare the structure of ant-plant mutualist networks in Continuous Forest (CF) and Forest Fragments (FF) and results of statistical analyses.

Metric	H_0_	Calculation	Pooled results		Fragment-level statistical comparisons		
			CF	FF	Mean_CF_±SEM	Mean_FF_±SEM	Pr>F
Number of ant species	1	ant species	10	7	6.5±0.87	4.5±0.5	0.092
Number of plant species	1	plant species	11	7	7.25±0.75	4.25±0.48	**0.015**
Links per species	2	links/species	1	1.21	0.9±0.04	1.02±0.09	0.295
Connectance	2	links/species	0.19	0.35	0.279±0.04	0.496±0.09	0.074
Linkage Density^1^	2	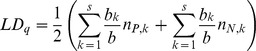	1.8	1.8	1.732±0.05	1.648±0.07	0.351
Nestedness^2^	3	With BINMATNEST in Bipartite (51)	35.98	33.26	43.32±3.4	25.85±8.5	0.105
Weighted Nestedness^3^	3	WINE (53)	0.06	0.13	0.188±0.07	0.103±0.07	0.421
Robustness (ants)	4	(33)	0.56	0.56	0.483±0.05	0.393±0.05	0.253
Robustness (plants)	4	(33)	0.54	0.57	0.464±0.03	0.533±0.08	0.440

1 See (10) for a complete description. s = number of species in the web and *b_•k_* and *b_k•_* represent column sum and row sums, respectively, of the plant/ant matrix, i.e., the total number of individuals associated with taxon k.

2 Nestedness ranges from 0 to 100, with 0 being most nested and 100 indicating complete randomness.

3 Weighted nestedness ranges from 0 to 1, where 1 is the most nested.

To determine if ant and plant species richness was lower in fragments (Hypothesis 1), we compared the number of ant and plant species in the networks. We then tested whether there were fewer realized interactions between ants and plants in fragments (i.e., connectance is higher in fragments, Hypothesis 2) by comparing the number of links per species, network connectance (both qualitative), and linkage density (quantitative) for each treatment with separate one-way ANOVAs. Because there is the potential for correlation among different metrics of network structure [39], we used a Bonferroni-adjusted alpha of 0.025 to assess the significance of the two qualitative measures. Next, to determine whether nestedness was similar between forest types (Hypothesis 3) we calculated the quantitative and qualitative versions of the network nestedness metric (Table 1) and again compared the treatments with one way-ANOVA.

Finally, to test if networks in fragments and continuous forest were equally resilient to disturbance (Hypothesis 4) we calculated the network robustness (R) to simulated extinction of ant or plant species (Table 1). The quantitative measure of robustness, R, is the area under the extinction curve (the attack tolerance curve or ATC, sensu Memmott *et al.* [27]; species are removed based on abundance with the least abundant species going extinct first) and can be calculated separately for both groups of mutualists. An R value of 1 indicates a very robust system, while a value of 0 indicates a fragile one [27], [40].

## Results

We censused N = 322 myrmecophytic plants in forest fragments and N = 653 in continuous forest; for a detailed description of these results see [31]. Summaries of the number of individual ant-plant interactions observed in continuous forest and forest fragments are in Tables 2 and 3, respectively; data on the frequency of interactions in individual plots are in [41].

**Table 2:** Matrix of the frequency of each obligate ant-myrmechophyte interaction observed in continuous forest (all plots combined).

Continuous Forest												
	Ants^1^	1	2	3	4	5	6	7	8	9	10	U^2^
		Ad	Ao	As	Az	Cab	Cl	Me	Pc	Pn	Pm	
**Plants** 3												
**1**	Cp					1						6
**2**	Cn		1		39							96
**3**	Ds		2	5	1							2
**4**	Hm	8	220	81								62
**5**	Hp		23	10	3							2
**6**	Mg										1	1
**7**	Mm							10				1
**8**	Pr		1									
**9**	Tm				5				26	11		9
**10**	Tp											
**11**	Tv								10			
**12**	Tb				9		3					4

**Figure 1:**
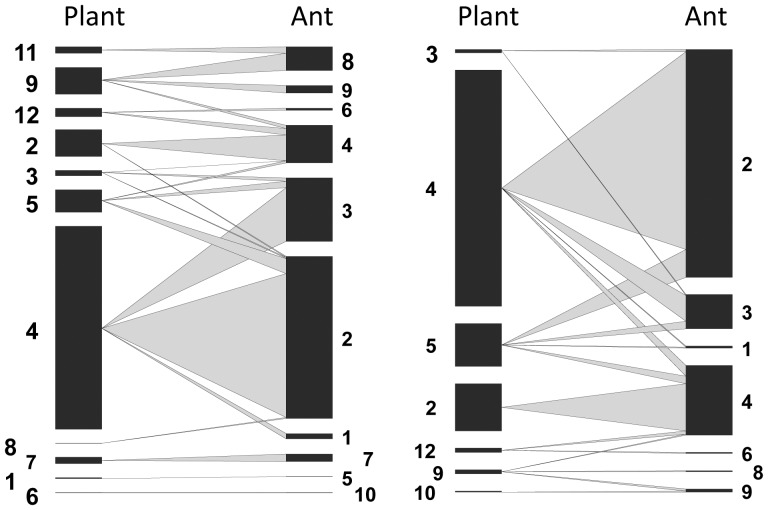
Networks for Continuous Forest (left) and Forest Fragment (right) based on data pooled across all sites. For each network vertical bars on the left represent plant abundance and bars on the right represent ant abundance; the width of the grey lines connecting them represents the frequency of that interaction. Ant species: 1 *Allomerus decemarticulatus*, 2 *Allomerus octoarticulatus*, 3 *Allomerus septemarticulatus*, 4 *Azteca* spp., 5 *Camponotus balzani*, 6 *Crematogaster laevis*, 7 *Myrcidris epicharis*, 8 *Pseudomyrmex concolor*, 9 *Pseudomyrmex nigrescens*, 10 *Pheidole minutula*. Plant species: 1 *Cecropia purpurascens*, 2 *Cordia nodosa*, 3 *Duroia saccifera*, 4 *Hirtella myrmecophila*, 5 *Hirtella physophora*, 6 *Maieta guianensis*, 7 *Myrcia madida*, 8 *Porouma* spp., 9 *Tachigali myrmecophila*, 10 *Tachigali pumblea*, 11 *Tachigali venusta*, 12 *Tococa bullifera*.

**Figure 2:**
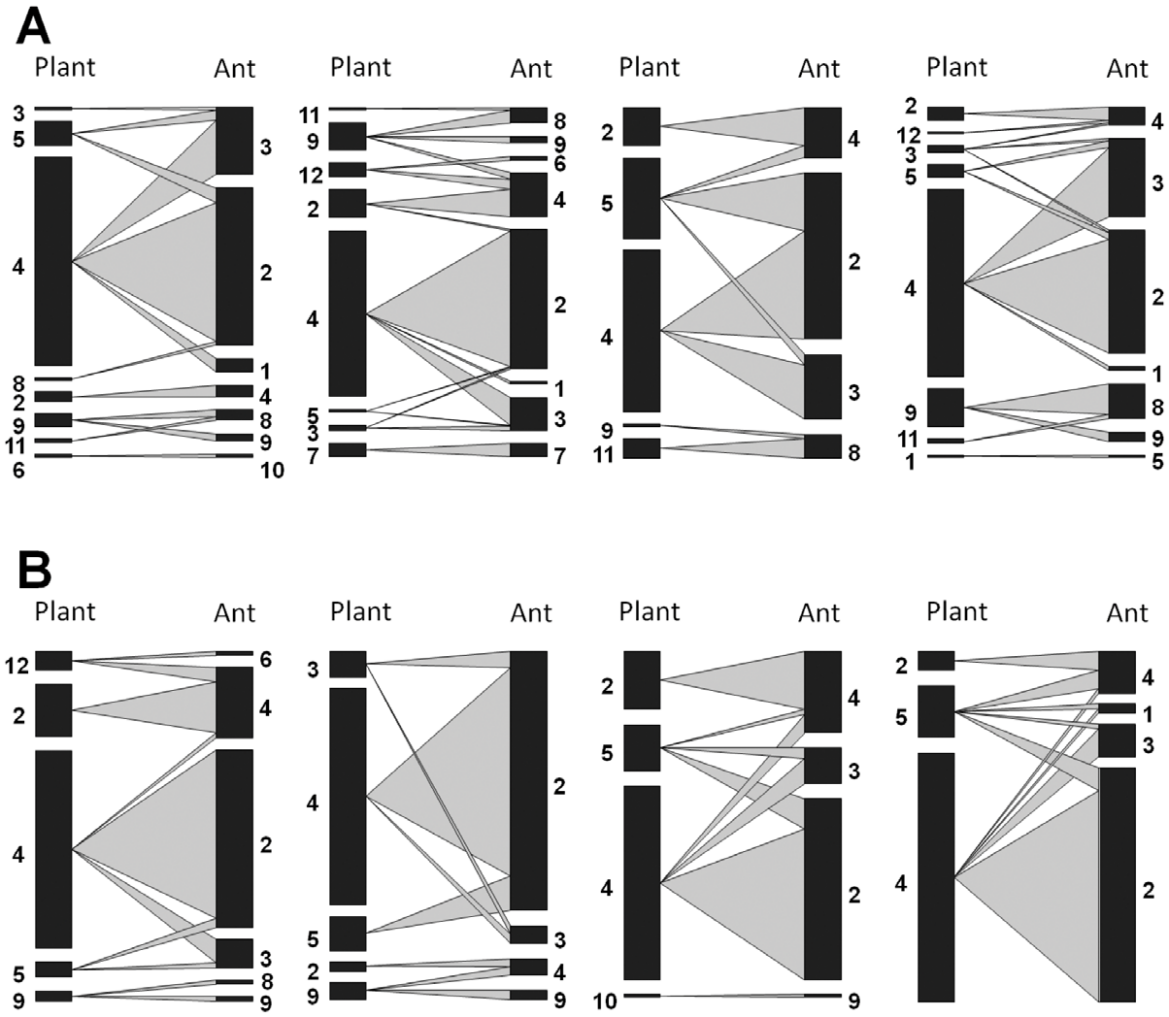
Ant-plant networks for individual plots. For each network, the bars on left represent plant species and bars on the right represent ant species; the width of the grey lines connecting them represents the frequency of that interaction. (A) Plots in Continuous Forest (CF), from left to right: “Camp 41”, “Dimona”, “Florestal” and “Porto Alegre”. (B). Plots in Forest Fragments (FF), from left to right: “Porto Alegre”, “Colosso”, “2108” and “2107”. See Bruna *et al.* [31] for the location and description of these sites; for a key to the plant and ant species see Figure 1.

Numbers in bold are the same as those used to identify species in Figures 1 and 2. Note that unoccupied plants (U) of all species were primarily seedlings [31].

1 Ant species: 1 *Allomerus decemarticulatus* (Ad), 2 *Allomerus octoarticulatus* (Ao), 3 *Allomerus septemarticulatus* (As), 4 *Azteca* spp. (Az), 5 *Camponotus balzani* (Cab), 6 *Crematogaster laevis* (Cl), 7 *Myrcidris epicharis* (Me), 8 *Pseudomyrmex concolor* (Pc), 9 *Pseudomyrmex nigrescens* (Pn), 10 *Pheidole minutula* (Pm).

2 Unoccupied plants (U).

3 Plant species: 1 *Cecropia purpurascens* (Cp), 2 *Cordia nodosa* (Cn), 3 *Duroia saccifera* (Ds), 4 *Hirtella myrmecophila* (Hm), 5 *Hirtella physophora* (Hp), 6 *Maieta guianensis* (Mg), 7 *Myrcia madida* (Mm), 8 *Porouma* spp. (Pr), 9 *Tachigali myrmecophila* (Tm), 10 *Tachigali pumblea* (Tp), 11 *Tachigali venusta* (Tv), 12 *Tococa bullifera* (Tb).

**Table 3:** Matrix of the frequency of each obligate ant-myrmechophyte interaction observed in forest fragments (all plots combined).

Forest Fragments												
	Ants^1^	1	2	3	4	5	6	7	8	9	10	U^2^
		Ad	Ao	As	Az	Cab	Cl	Me	Pc	Pn	Pm	
**Plants** 3												
**1**	Cp											2
**2**	Cn				39							14
**3**	Ds		2	1								
**4**	Hm	1	164	22	9							10
**5**	Hp	1	23	6	6							1
**6**	Mg											1
**7**	Mm											
**8**	Pr											4
**9**	Tm				1				1	2		3
**10**	Tp									1		
**11**	Tv											2
**12**	Tb				3		1					2

Numbers in bold are the same as those used to identify species in Figures 1 and 2. Note that unoccupied plants of all species were primarily seedlings [31].

1 Ant species: 1 *Allomerus decemarticulatus* (Ad), 2 *Allomerus octoarticulatus* (Ao), 3 *Allomerus septemarticulatus* (As), 4 *Azteca* spp. (Az), 5 *Camponotus balzani* (Cab), 6 *Crematogaster laevis* (Cl), 7 *Myrcidris epicharis* (Me), 8 *Pseudomyrmex concolor* (Pc), 9 *Pseudomyrmex nigrescens* (Pn), 10 *Pheidole minutula* (Pm).

2 Unoccupied plants (U).

3 Plant species: 1 *Cecropia purpurascens* (Cp), 2 *Cordia nodosa* (Cn), 3 *Duroia saccifera* (Ds), 4 *Hirtella myrmecophila* (Hm), 5 *Hirtella physophora* (Hp), 6 *Maieta guianensis* (Mg), 7 *Myrcia madida* (Mm), 8 *Porouma* spp. (Pr), 9 *Tachigali myrmecophila* (Tm), 10 *Tachigali pumblea* (Tp), 11 *Tachigali venusta* (Tv), 12 *Tococa bullifera* (Tb).

We found that networks in continuous forest had significantly more plant species than networks in forest fragments (N = 7.25 vs. N = 4.25, respectively, P = 0.015, Figure 1, Table 1), and there was a trend towards more ant species in continuous forests than forest fragments (N = 10 vs. N = 7, respectively, P = 0.092). However, none of the other eight metrics we calculated differed for networks in forest fragments and continuous forest (Table 1, Table S1). On average species were linked to approximately one other species, though there was a trend towards lower connectance in continuous forests than forest fragments (0.279±0.04 vs. 0.496±0.09, respectively, p = 0.351). Nestedness was, on average, 43.32±3.4 in forest fragments and 25.85±8.5 in continuous forest. Summary networks (Figure 2) had values for all metrics that were similar to the averages for plots in that habitat class (Table 1).

## Discussion

Tropical forest fragments undergo myriad biotic and abiotic changes following their isolation, among the most notable of which are the local extinctions of plant and animal species [17], [42], [43]. Because most plant and animal species are involved in mutualisms, it has been suggested that the extinctions of individual taxa could result in a “cascade of extinctions” reverberating throughout communities of interacting taxa [44]. We found that the networks of ant-plant mutualists in twenty-five year old fragments are similar to those in continuous forest, suggesting these interactions are surprisingly resistant to the detrimental changes associated with habitat fragmentation. These results echo those of Kaartinen and Roslin's [45] comprehensive study of food webs associated with oak trees in Finland, in which landscape context had no detectable effect on interaction evenness, linkage density, connectance, or network vulnerability. However, generalities regarding the effect of habitat fragmentation on mutualistic networks clearly require additional studies, including those explicitly evaluating how network structure changes in fragments of different sizes and with a broader diversity of mutualistic interactions [9], [46].

It is also important to emphasize that ant-plant mutualistic networks have several unique properties that may promote their persistence in fragmented landscapes. Nested mutualistic networks are expected to be resilient to species losses if extinctions involve specialists that are involved in fewer interactions within the network [27], [40]. Synergisms between species rarity and habitat specialization were found to lead to extinction in beetle species in fragmented habitats [26]. This is probably why ant-plant networks in fragments and continuous forest were similar in structure despite species losses – the species lost in fragments tended to be those with only one connection. For instance, the ants *Myrcidris epicharis*, *Camponotus balzani*, and *Pheidole minutula* – each of which was linked to only one plant species in continuous forest – were all absent in fragments. Similarly, five plant species missing in fragments (i.e., *Tachigali venusta*, *Porouma* spp., *Myrcia madida*, *Cecropia purpurascens*, *Maieta guianensis*) were associated with only one ant species in continuous forest sites. Such associations formed isolated subwebs, which are common in symbiotic interaction networks [30], [47], that were completely absent in the pooled fragment networks (although 3 of 4 of the individual plot networks in forest fragments also had subwebs, see Figure 2). The loss of specialists from a nested network is expected to have little effect on the overall structure of the network [27], [40]; in this case the nested structure may have reduced the transmission of disturbance through the rest of the community [4], [48].

In general, it appears that the resilience of mutualistic networks is enhanced by higher species richness, network connectivity, and through strong, symmetric interaction within highly nested networks [49], [50]. Our results provide additional evidence in support of one additional mechanism promoting the persistence of mutualist networks following disturbance - symbiosis. Mutualisms that are symbiotic, such as those between ants and myrmecophytic plants [47], appear more resilient to disturbances than networks of non-symbiotic mutualistic interactions (e.g., seed dispersal, pollination). Because symbiotic networks tend to have lower species richness and more isolated subwebs compared to non-symbiotic networks, the loss of a single partner species rarely reverberates throughout the community [47]. In light of the challenges in conducting manipulations of biodiversity in a field setting, tests of this hypothesis would benefit from the rigorous integration of models and empirical studies advocated by Morris [8].

Although our study was not designed to compare within-habitat variation in the structure of networks, it appears that replicates within a single habitat class were often quite variable. Within habitat heterogeneity is hypothesized to have a major influence on network topology [11], [51] via sampling effects [35] and because interaction probabilities will depend on the distribution of individuals across the landscape [11]. Unfortunately, most studies of mutualistic networks have been conducted in a single location [52], so addressing this important issue remains a challenge. In our sites, common species were consistently present but links with less common species were unpredictable. Given that the effects of fragment size, fragment isolation, and species loss can be confounded, mesocosm studies [9] could provide a useful complementary tool for identifying the causal factors by which landscape structure influences network structure.

Three important caveats to our results bear consideration. First, the taxonomies of the ant genus *Azteca* remains challenging and has yet to be fully resolved for our study sites [30], [53]. Consequently, we have under-estimated the number of species involved in the ant-plant network by pooling multiple *Azteca* species in a single taxon for our analyses. Our ongoing molecular analyses are attempting to determine the number of *Azteca* species colonizing myrmecophytes, and including multiple *Azteca* species species will clearly alter the structure of networks. However, the overall conclusion that networks are similar in forest fragments and continuous forests will likely remain unchanged – with the exception of *Cordia nodosa*, plant species colonized by *Azteca* are relatively rare [30] and have similar abundances in both fragments and continuous forest [31]. Second, our experimental fragments are relatively young compared with those in other locations, such as the Atlantic Forests of Brazil's northeast [54]. Because many of the plant species in our network are long-lived [55], it may yet be decades until changes in their abundance result in altered network structure. Finally, our results may be conservative because the BDFFP fragments are protected from fire, the incursion of cattle, and other forms of anthropogenic disturbance. In addition, there are large expanses of nearby primary and regenerating forest from which propagules of some ant and plant species could disperse into fragments, and the matrix in which our fragments are embedded may be much more permeable to dispersing species than other matrix types (e.g., active cattle pastures, sugarcane fields). On the other hand, these 1-ha fragments are extremely small and even their interiors are subjected to the most severe of abiotic edge effects [56]. Furthermore, recent work suggests the distance between fragments and nearby continuous forest may be sufficient to prevent the colonization of several common partner ant species [53], which may be why the density of the most common ant-plants in our study fragments is lower than in nearby continuous forest [31]. Finally, conducting our study in the BDFFP's experimental landscape allowed for us to minimize the effects of inter-fragment variability that often plague studies conducted in ‘naturally’ fragmented landscapes. However, it also meant our surveys were limited to the number of fragments available at the BDFFP. Though the environmental changes in our study fragments are often severe [53] and our results are remarkably consistent across different metrics of network structure, it is possible that small but significant differences would be detected with the power resulting from increasing the number of fragments sampled. A robust test of our conclusion that ant-plant networks are resistant to the effects of fragmentation will clearly require additional studies with larger sample sizes, across a range of fragment sizes and ages [13], and in landscapes where fragments are afforded different levels of protection from anthropogenic impacts.

We conclude that the redundancy built into mutualistic networks and the limited number of 1∶1 interactions in tropical ant-plant systems makes these networks inherently resistant to the effects of fragmentation, at least in the short term [45]. However, ongoing deforestation and climate change continue to influence species distributions worldwide, and may thus influence the structure of networks in unexpected ways [57], [58], [59]. By proactively identifying key species in webs (e.g., the well-connected *Allomerus octoarticulatus* and *Hirtella myrmecophila* in our network), it may be possible to focus conservation efforts on those species in addition to the more commonly targeted rare or endemic ones as a means of ensuring the integrity of network structure and ecosystem services these networks provide [8], [9], [15].

## Supporting Information

Table S1 Tables for one-way ANOVAs of nine network metrics. The metric with a significant p-value (alpha level = 0.05) is in bold.
(DOC)
