# Supplementary material for: Resilient Networks of Ant-Plant Mutualists in Amazonian Forest Fragments
Source: PLoS One. 2012 Aug 9;7(8):e40803. doi: 10.1371/journal.pone.0040803 (PMC3415396; doi:10.1371/journal.pone.0040803)
Supplement: Table S1 — Tables for one-way ANOVAs of nine network metrics. The metric with a significant p-value (alpha level = 0.05) is in bold. (DOC) [file pone.0040803.s001.doc]

**SUPPLEMENTAL MATERIALS**

**Table S1.** Tables for one-way ANOVAs of eleven network metrics. Two metrics with significant p-values (alpha level = 0.05) are in bold.

**Number of Ant Species**

Source DF SS MS F-ratio Prob>F

Habitat 1 8.0 8.0 4.0 0.0924

Error 6 12.0 2.0

Total (corrected) 7 20.0

**Number of Plant Species**

Source DF SS MS F-ratio Prob>F

Habitat 1 18.0 18.0 11.3684 **0.0150**

Error 6 9.5 1.58

Total (corrected) 7 27.5

**Links per Species**

Source DF SS MS F-ratio Prob>F

Habitat 1 0.0266 0.0266 1.3134 0.2954

Error 6 0.1216 0.0203

Total (corrected) 7 0.1482

**Connectance**

Source DF SS MS F-ratio Prob>F

Habitat 1 0.0944 0.0944 4.6922 0.0735

Error 6 0.1207 0.0201

Total (corrected) 7 0.2151

**Linkage Density**

Source DF SS MS F-ratio Prob>F

Habitat 1 0.0143 0.0143 1.0231 0.3508

Error 6 0.0841 0.0140

Total (corrected) 7 0.0985

**Nestedness**

Source DF SS MS F-ratio Prob>F

Habitat 1 610.7 610.7 3.6401 0.1050

Error 6 1006.7 167.8

Total (corrected) 7 1617.4

**Weighted Nestedness**

Source DF SS MS F-ratio Prob>F

Habitat 1 0.0145 0.0145 0.7452 0.4212

Error 6 0.1164 0.0194

Total (corrected) 7 0.1308

**Robustness (ants)**

Source DF SS MS F-ratio Prob>F

Habitat 1 0.0164 0.0164 1.5965 0.2533

Error 6 0.0616 0.0103

Total (corrected) 7 0.0779

**Robustness (plants)**

Source DF SS MS F-ratio Prob>F

Habitat 1 0.0097 0.0097 0.6824 0.4404

Error 6 0.0857 0.0143

Total (corrected) 7 0.0954
